# Hemophagocytic Syndrome-Associated Intravascular Large B-cell Lymphoma With Dialysis-Dependent End-Stage Renal Disease Treated With Autologous Stem Cell Transplantation Using a Modified TEAM Regimen

**DOI:** 10.7759/cureus.25885

**Published:** 2022-06-12

**Authors:** Kudret Kama, Paul La Rosée, David Czock, Jan Bosch-Schips, Gerald Illerhaus

**Affiliations:** 1 Department of Hematology, Oncology and Palliative Care, Klinikum Stuttgart, Stuttgart, DEU; 2 Clinic for Internal Medicine II, Schwarzwald-Baar Klinikum, Villingen-Schwenningen, DEU; 3 Department of Clinical Pharmacology and Pharmacoepidemiology, University Hospital Heidelberg, Heidelberg, DEU; 4 Institute of Pathology and Neuropathology and Comprehensive Cancer Center Tübingen, University Hospital Tübingen, Tübingen, DEU

**Keywords:** stem cell transplantation, team regimen, end-stage renal disease, hlh, intravascular large b-cell lymphoma

## Abstract

Due to the low incidence and the large number of postmortem diagnoses, treatment recommendations for intravascular large B-cell lymphoma (IVLBCL) are largely based on retrospective studies and case reports. There is little data on autologous stem cell transplantation (ASCT) in dialysis-dependent patients and choosing an adequate regimen and dosing is difficult. Here, we report the treatment of a patient with relapsed IVLBCL and end-stage renal disease caused by lymphoma-associated renal AA amyloidosis using a modified TEAM (thiotepa, etoposide, cytarabine, and melphalan) regimen and ASCT. A 42-year-old female had an early relapse of hemophagocytic syndrome-associated intravascular large B-cell lymphoma resulting in terminal renal disease with dialysis dependency. Because of comorbidities (AA amyloidosis with severe hypoalbuminemia and end-stage renal disease), a modified, dose-reduced TEAM regimen was used as a high-dose conditioning regimen based on clinical pharmacologic considerations. The patient developed grade three mucositis and grade four febrile neutropenia as adverse events after transplantation. A modified TEAM regimen is feasible in a patient with end-stage renal disease with manageable toxicity. This is the first report of treatment with thiotepa in a dialysis-dependent patient.

## Introduction

Intravascular large B-cell lymphoma (IVLBCL) is a rare disease characterized by the proliferation of lymphoma cells within the lumina of small vessels. Even though early diagnosis and treatment are vital, the diagnosis is often delayed due to a variety of possible symptoms and the lack of detectable tumor masses. The new WHO classification for neoplasms and lymphomas distinguishes between two variants of IVLBCL [1]: the classical variant, formerly the “Western variant,” presents with cutaneous involvement at diagnosis in 43% of patients and has a three-year overall survival of up to 81%, and a variant associated with the hemophagocytic syndrome, formerly “Asian variant” [2]. The hemophagocytic syndrome-associated variant has an aggressive clinical course with involvement of the liver, spleen, and pancytopenia. The median survival time is less than eight months in these patients. Intensive Rituximab (R)-CHOP (cyclophosphamide, doxorubicin, vincristine, and prednisone)-like immunochemotherapy is currently the standard of care [3].

Treating a rare disease like intravascular lymphoma is challenging. Due to the low incidence and large number of postmortem diagnoses, treatment recommendations for IVLBCL are largely based on retrospective studies and case reports. Standard R-CHOP achieves a complete remission rate of up to 90%, but the relapse rate is very high with a two-year progression-free survival (PFS) of only 56% [3-4]. A recent study, the first prospective phase two study in IVLBCL, demonstrated that R-CHOP with high-dose methotrexate and intrathecal chemotherapy can improve two-year PFS to 76% [5]. There is little data on autologous stem cell transplantation (ASCT) in dialysis-dependent patients and choosing an adequate regimen and dosing is difficult.

Here, we report the treatment of a patient with relapsed IVLBCL and end-stage renal disease caused by lymphoma-associated renal AA amyloidosis using a modified TEAM (thiotepa, etoposide, cytarabine, and melphalan) regimen and ASCT.

This article was previously presented as a meeting abstract at the 2020 DGHO Meeting on October 09, 2020.

## Case presentation

**Figure 1:**
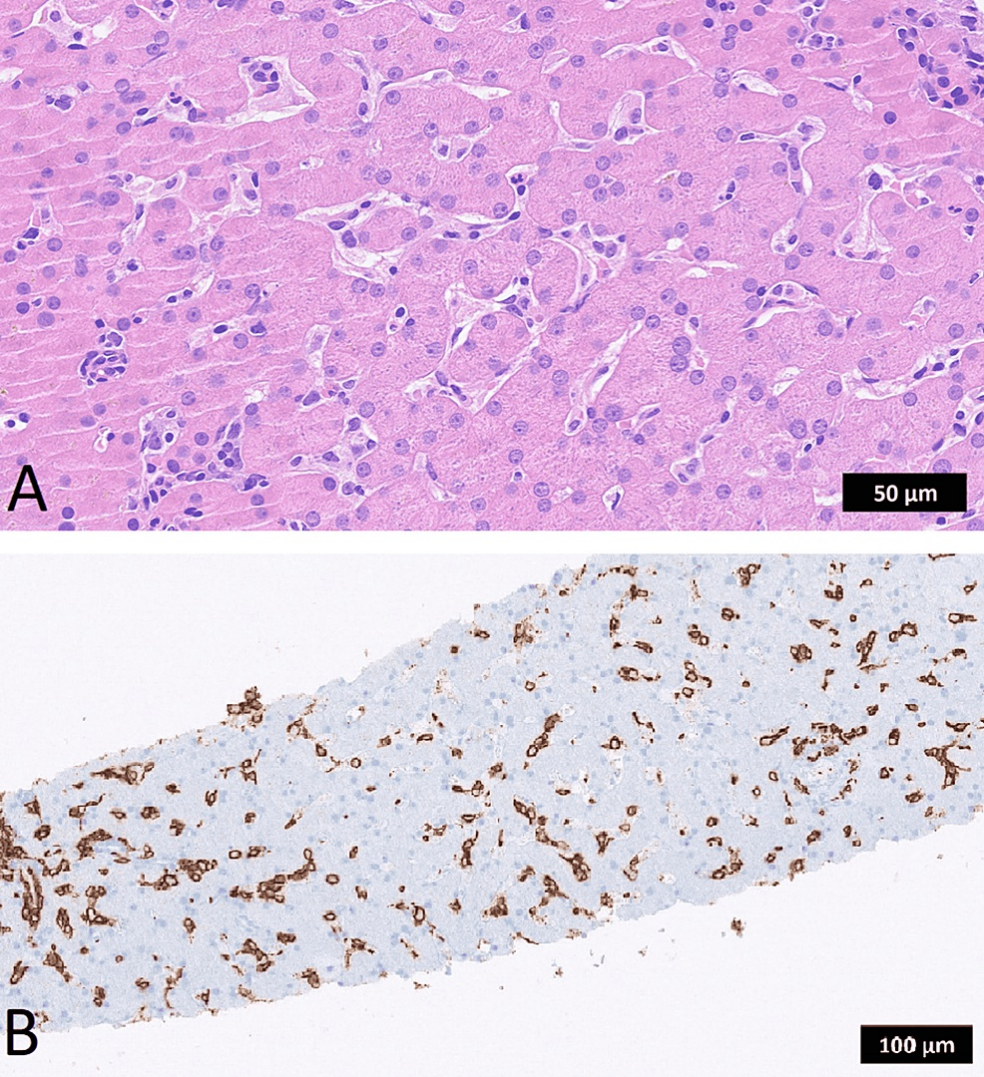
Histopathological features of IVLBCL in liver biopsy (A) Intermediate-sized atypical lymphoid cells within minimally distended hepatic sinusoids. Tumor cells exhibited scant cytoplasm and hyperchromatic nuclei with irregular contours (Hematoxylin and Eosin [H&E]; original magnification, x400). (B) Neoplastic cells highlighted by strong CD20 expression, confirming B-cell origin (immunoperoxidase; original magnification, x200). IVLBCL: intravascular large B-cell lymphoma

A 42-year-old female presented with fever, hepatosplenomegaly, and pancytopenia. She was diagnosed with hemophagocytic lymphohistiocytosis (HLH) due to fulfilling six (fever, splenomegaly, bicytopenia, hypertriglyceridemia, high soluble interleukin-2-receptor (sIL-2R), and hemophagocytosis) of the eight diagnostic criteria [6]. Extensive diagnostic workup, including wide-spectrum infectious disease tests, could not reveal an underlying disease. With ongoing clinical deterioration, treatment according to the HLH-94 protocol (six doses of etoposide 150 mg/m² and dexamethasone 10 mg/m^2^ over four weeks) was begun [6]. No clinical benefit but ongoing pancytopenia, fever, and progressive hepatosplenomegaly were observed. After four weeks of treatment, the patient developed severe hypoalbuminemia and a renal biopsy confirmed AA amyloidosis (SAA-alpha-mutation positive). Because of rising serum sIL-2R levels (≥13,000 U/ml) and a high sIL-2R/ferritin ratio (> 4.6), an occult lymphoma was suspected [7]. The patient was then treated with six cycles of R-CHOP and achieved a complete remission. After four months, an early relapse of HLH occurred resulting in acute renal failure and dialysis dependency. A random liver biopsy was performed and immunohistochemical studies revealed strong CD20 expression and positivity for CD5, BCL2, CD10 (focal), and BCL6 (germinal center B-cell type). Tumor cells were negative for CD3 and MUM1, and MYC protein was not significantly expressed (<40%). These features were consistent with IVLBCL, and the patient was diagnosed with infiltration of the liver by an IVLBCL with co-expression of CD5, as seen in Figures 1A-1B.

As salvage treatment, she received R-GDP (rituximab 375 mg/m^2^ on Day 0, gemcitabine 1000 mg/m^2^ on Day 2 and Day 9, dexamethasone 40 mg on Day 1 to 4, and cisplatin 75 mg/m^2^ on Day 1) for five cycles resulting in a partial response. Stem cells were successfully harvested after the third cycle. Because of comorbidities (AA amyloidosis with severe hypoalbuminemia and end-stage renal disease), a modified, dose-reduced TEAM regimen was used as a high-dose conditioning regimen based on clinical pharmacologic considerations. The TEAM regimen consisted of thiotepa 5 mg/kg on Day -7, etoposide 100 mg/m^2^, and cytarabine 200 mg/m^2^ once daily on Days -6 to -3, and melphalan 100 mg/m^2^ on Day -2, as seen in Table 1.

**Table 1:** Modified TEAM regimen before ASCT Thiotepa, cytarabine, and etoposide were administered 12 hours before dialysis, and melphalan was administered after dialysis. Due to severe hypoalbuminemia, albumin was substituted daily during hemodialysis starting one day prior to conditioning. TEAM: thiotepa, etoposide, cytarabine, and melphalan; ASCT: autologous stem cell transplantation

Therapy	Dose Reference	Case	Day -8	Day -7	Day -6	Day -5	Day -4	Day -3	Day -2	Day -1	Day 0	Day +1
Thiotepa (presumably dialyzable)	5 mg/kg	5 mg/kg		x								
Etoposide (not dialyzable)	100 mg/m^2^ (twice daily)	100 mg/m2 (once daily)			x	x	x	x				
Cytarabine (dialyzable)	200 mg/m^2^ (twice daily)	200 mg/m2 (once daily)			x	x	x	x				
Melphalan (dialyzable)	140 mg/m^2^	100 mg/m2							x			
ASCT											x	
Hemodialysis				x	x	x	x	x	x	x		x
Albumin substitution		20%, 200 ml	x	x	x	x	x	x	x	x		x

Intermittent hemodialysis with a high‐flux dialysis membrane was performed every day over four hours in order to eliminate excessive active or toxic metabolites.

**Figure 2:**
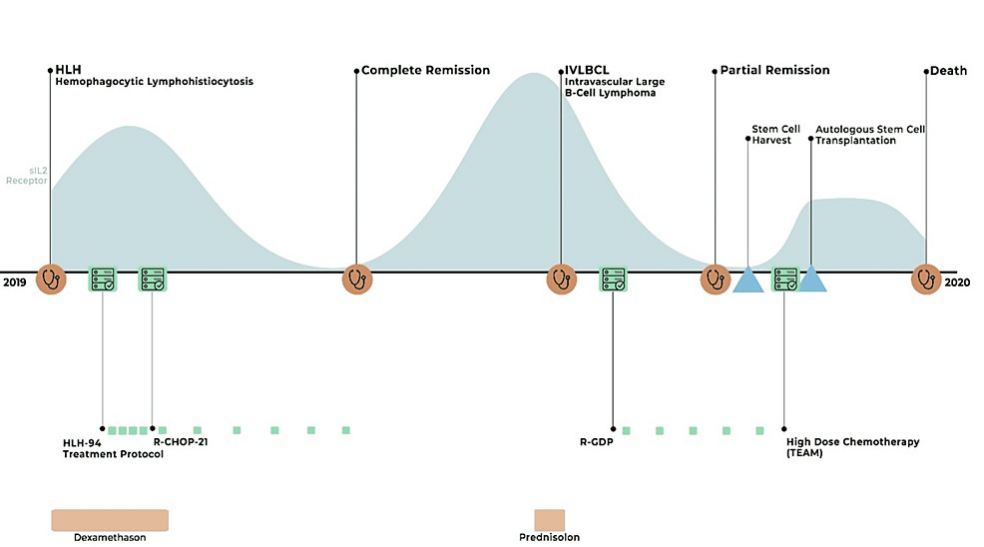
Timeline

No acute toxicity was observed after administration of high-dose chemotherapy. 2.94 x 10^6^ CD34^+^ cells/kg were successfully transplanted. The patient developed grade three mucositis and grade four febrile neutropenia as adverse events after transplantation. Neutrophil engraftment was observed on day 16, and the time to platelet engraftment was 39 days. On Day 40, the sIL-2R level was elevated with progressive hepatosplenomegaly, but a bone marrow aspirate and a second liver biopsy showed no indication of lymphoma or hemophagocytosis. After more than a year of intensive treatments, the patient wished for an end to further therapy and dialysis. We respected the patient´s wish and proceeded with the best supportive care at our palliative unit. The patient died on Day 80 after ASCT. The clinical course of the patient can be seen in Figure 2.

## Discussion

A diagnostic pitfall was the failure to obtain histopathologic confirmation of the underlying lymphoma by blind liver biopsy at initial presentation. If an IVLBCL is suspected, a blind skin and liver biopsy are recommended [1]. The time to definite diagnosis was nine months, as IVLBCL was not diagnosed before relapse. Protracted treatment may have paved the way for AA amyloidosis and subsequent terminal renal disease with dialysis dependency. As a result, optimal dosing of high-dose chemotherapy was complicated, and an allogeneic stem cell transplantation was no longer an option.

Consolidation should have been discussed after achieving complete remission with R-CHOP. Particularly in patients with the hemophagocytic variant, high-dose chemotherapy and ASCT should be considered in the first-line treatment of IVLBCL due to the high relapse rate [4].

As salvage therapy, R-GDP was chosen, and no adaptations were made to the regimen because all agents can be administered in dialysis-dependent patients. Cisplatin is dialyzable and dialysis was performed shortly after each cisplatin infusion [8]. No grade three or four adverse events were seen after five cycles of R-GDP. In a retrospective study evaluating ASCT in IVLBCL, most patients received BEAM (carmustine, etoposide, cytarabine, and melphalan) as the conditioning regimen with a two-year overall survival of 91% [9]. For conditioning therapy, we decided to use a modified TEAM regimen instead of BEAM due to the risk of acute and late toxicities of carmustine [10].

In dialysis-dependent patients, finding the optimal dosing for conditioning therapy before transplantation is difficult due to the risk of overdosing and increased toxicity or underdosing, and insufficient disease control. Dose adaptations were made according to reports regarding the pharmacokinetics of these agents. Most data for use as a conditioning regimen exist for melphalan, as it is used frequently in dialysis-dependent multiple myeloma patients with a recommended dose of 100 mg/m^2 ^[11]. The cytarabine metabolite uracil arabinoside (Ara-U) accumulates in patients with renal failure and is suspected to explain the increased cytarabine neurotoxicity in patients with renal impairment [12]. Thus, the cytarabine dose was reduced to 200 mg/m^2^ once daily, which equals a dose reduction of 50%. Etoposide is not eliminated by hemodialysis, however, in a case series, a 50% dose reduction had good efficacy without additional adverse events in patients receiving dialysis [13].

While there are no reports of thiotepa in patients receiving dialysis, there is one case report about the pharmacokinetics of thiotepa in a patient with moderate renal insufficiency. Exposure to TEPA (N,N',N"-triethylenephosphoramide), the main and active metabolite of thiotepa with similar alkylating properties, was increased by about 150% with the split application over four days. Exposure of other metabolites (monochloro TEPA, thioTEPA mercapturate) was not quantified [14]. The pharmacokinetics of thiotepa is dose-dependent and with normal renal function, less than 1% of unchanged thiotepa is found in the urine. Approximately 10% of the administered dose is recovered in urine as TEPA or as thioTEPA mercapturate, respectively, whereas recovery of monochloro TEPA was less than 1% [15]. Given the known pharmacokinetics of thiotepa and its metabolites (low molecular weight, low protein binding, moderate apparent volume of distribution), we assumed that thiotepa could likely be eliminated by hemodialysis and no dose reductions were made. With a typical half-life of 2.3 hours, most of the administered thiotepa should be eliminated after 12 hours. The time with alkylating activity is probably longer due to the TEPA half-life of approximately 10 hours [15]. Therefore, the first dialysis was set to 12 hours after thiotepa infusion in order to allow for (presumably) normal thiotepa exposure and to limit potentially prolonged exposure to active thiotepa metabolites. The main adverse events of thiotepa are mucositis and temporary central nervous system toxicity, and our patient developed grade three mucositis which resolved quickly [14].

The hematological recovery after ASCT was delayed, which could be caused by prolonged fever after transplantation or increased toxicity by overdosing. We could not confirm relapse before death but had a strong suspicion due to elevated serum sIL-2R.

## Conclusions

The presented case illustrates that a modified TEAM regimen is feasible in a patient with end-stage renal disease with manageable toxicity. To our knowledge, this is the first report of thiotepa in a dialysis-dependent patient. Due to the rapidly progressive and fatal course of IVLBCL, it is essential to aim for aggressive, i.e. blind multiorgan biopsy particularly in patients, where HLH-associated lymphoma is suspected. Early, aggressive treatment with R-CHOP or R-CHOEP and high-dose chemotherapy with ASCT should be considered in high-risk and eligible patients.
